# Gut microbiota and parasite dynamics in an Amazonian community undergoing urbanization in Colombia

**DOI:** 10.1128/msphere.00788-25

**Published:** 2026-01-28

**Authors:** Sebastián Díaz, Amie J. Eisfeld, Mónica Palma-Cuero, Nathalie Dinguirard, Leah A. Owens, Karl A. Ciuoderis, Laura S. Pérez-Restrepo, John D. Chan, Tony L. Goldberg, Jessica L. Hite, Juan Pablo Hernandez-Ortiz, Yoshihiro Kawaoka, Mostafa Zamanian, Jorge E. Osorio

**Affiliations:** 1 UW-GHI One Health Colombia, Universidad Nacional de Colombia, Medellín, Colombia; 2 Department of Pathobiological Sciences, University of Wisconsin-Madison 5228, Madison, Wisconsin, USA; 3 Laboratorio de Salud Pública Departamental del Amazonas, Grupo de Estudios en Salud Pública de la Amazonía, Leticia, Colombia; 4 Global Health Institute, University of Wisconsin-Madison 5228, Madison, Wisconsin, USA; 5 Corporacion Corpotropica, Villavicencio, Colombia; 6 Faculty of Life Sciences, Universidad Nacional de Colombia 124148, Medellín, Colombia; University of Michigan Medical School, Ann Arbor, Michigan, USA

**Keywords:** Colombia, protozoa, Amazon, helminths, microbiome

## Abstract

Studies on human gut microbiota have recently highlighted a significant decline in bacterial diversity associated with urbanization, driven by shifts toward processed diets, increased antibiotic usage, and improved sanitation practices. This phenomenon has been largely overlooked in the Colombian Amazon, despite rapid urbanization in the region. In this study, we investigate the composition of gut bacterial microbiota and intestinal protozoa and soil-transmitted helminths (STHs) in both urban and rural areas of Leticia, located in the southern Colombian Amazon. Despite their geographic proximity, the urban population is predominantly non-indigenous, while indigenous communities mostly inhabit the rural area, resulting in notable lifestyle differences between the two settings. Our analyses reveal a reduction in bacterial families linked to non-processed diets, such as *Lachnospiraceae*, *Spirochaetaceae*, and *Succinivibrionaceae*, in the urban environment compared to their rural counterparts. *Prevotellaceae*, typically associated with non-processed food consumption, shows a significantly higher abundance in urban Leticia. STH infections were primarily detected in rural Leticia, while intestinal protozoa were ubiquitous in both rural and urban areas. Both types of parasites were associated with higher gut bacterial richness and diversity. Additionally, microbial metabolic prediction analysis indicated differences in pathways related to unsaturated fatty acid production and aerobic respiration between rural and urban bacterial microbiomes. This suggests a tendency toward changes in the urban microbiota that may lead to increased susceptibility to non-communicable chronic diseases. These findings provide new insights into the impact of urbanization on gut microbiota dynamics in the Amazonian context and underscore the need for further research into any associated health outcomes.

**IMPORTANCE:**

Changes in the diversity and composition of gut microbiota in urban populations have been linked to the rise of non-communicable chronic diseases, such as autoimmune conditions, diabetes, and cancer. As developing countries undergo a demographic shift toward increased urbanization, accompanied by changes in diet, housing, and medication use, there is a concerning loss of microbial diversity. Therefore, it is essential to investigate microbiota changes in overlooked populations, such as indigenous communities in the Colombian Amazon basin. A better understanding of local and generalizable changes in gut microbial composition through urbanization may facilitate the development of targeted programs aimed at promoting lifestyle and diet changes to prevent diseases that healthcare systems may be ill-equipped to effectively address.

## INTRODUCTION

The taxonomic composition of the human gut microbiota is dynamic and shaped by factors such as diet, medication, sanitation, and occupational exposures (1). Surveys on microbiota composition during the rural-to-urban population transition have consistently observed a reduction in microbial diversity, particularly of bacterial groups associated with traditional lifestyles. These are known as VANISH (volatile and/or negatively associated with industrialized human societies) taxa. Examples include *Prevotellaceae*, *Spirochaetaceae*, and *Succinivibrionaceae* (2–5), whose decline is attributed to dietary shifts toward processed foods, widespread antibiotic usage, and decreased physical and outdoor activity. This is accompanied by a corresponding expansion of BloSSUM (bloom or selected in societies of urbanization/modernization) taxa, such as *Bacteroidaceae* and *Verrucomicrobia*, which are linked to an increased incidence of chronic disease (6, 7). These trends are expected to increase with the global rise of urbanization, with over half of the world’s population currently residing in urban areas (8).

Gut bacteria also interact with commensal and parasitic eukaryotes in the human host, including protists and soil-transmitted helminths (STHs) (9, 10). These single-celled and multicellular organisms interact with the bacterial community and are associated with increased richness and diversity, or changes in the abundance of specific bacterial taxa (11–17). For example, abundance of the *Prevotella* genus of gut bacteria is positively associated with *Blastocystis* spp. and *Endolimax nana* protozoa (12), but negatively associated with the presence of *Entamoeba* (18). These interactions can also be clinically relevant, such as the negative correlation between the *Megasphaera* genus of bacteria and diarrheal symptoms during *Cryptosporidium* infection (19). Helminth parasites also alter host-microbiota interactions (20), with several well-known immunomodulatory effects of helminth infection requiring host microbiota (21, 22). Infection with *Trichuris muris* and *Heligmosomoides polygyrus* roundworms inhibits proinflammatory bacterial taxa while promoting colonization with protective *Clostridiales* species, and this relationship can be reversed with deworming (23). The relative abundance of *Clostridiales* also changes in individuals infected with hookworm (*Necator americanus*) following anthelmintic treatment (24). Both protozoa and helminth parasites also stimulate intestinal tuft cells (25, 26). These cells promote type II immunity and alter intestinal microbiota composition (27, 28) while also secreting signals that directly modulate parasite biology (29).

Demographic and cultural change in the Amazonian region is characterized by an urban expansion of existing riverside towns and the establishment of new peri-urban settlements driven by agriculture, extractive industries, and infrastructure development (30). However, gut microbiota studies have predominantly focused on rural horticulturists and hunter-gatherers, with limited attention given to urban populations (31–36). Leticia, situated in the southern Colombian Amazon, serves as the capital city of the Departamento del Amazonas and forms an urban complex straddling the borders of Brazil, Colombia, and Peru. The city’s population is estimated at 100,000 inhabitants, with distinct demographic characteristics observed between urban and peri-urban rural areas (37, 38). Mixed-race populations predominantly inhabit the urban area, while indigenous groups primarily populate rural communities settled in response to extractive booms (e.g., rubber and coca) and assimilation efforts such as missionary campaigns (39).

This study provides novel insights into the urban and rural gut microbiota and parasite communities of a Colombian Amazonian population. We aim to understand how social factors, medical history, and current infection with parasitic protozoa and STHs interact with the bacterial microbiota composition. Additionally, using metabolic pathways predictions, we explore the potential health implications of the bacterial taxonomic differences between urban and rural areas.

## MATERIALS AND METHODS

### Human subjects

Healthy volunteers were recruited in March 2021 from two field sites: (i) the peri-urban rural multi-ethnic indigenous community of Nimaira Naimeki Ibiri Kilómetro 11 (referred to as Km11) (*n* = 80) and (ii) within the urban city limits of Leticia (referred to as Leticia) (*n* = 20). Colombian Army and Air Force support facilitated operations at the Km11 field site. Before enrollment, consent was obtained from all participants. For individuals under 18 years old, formal written consent was provided by their parents or legal guardians. Before sampling, each participant completed a survey addressing socioeconomic and health status.

### Donor sampling

Enrolled participants were provided with a specialized kit for fecal specimen collection, along with verbal instructions for proper sample acquisition. Upon collection, fecal specimens were promptly stored in pack ice for transportation to the State Public Health Laboratory. Aliquots of 1 g of the specimens were preserved in 2 mL of DNA/RNA Shield (Zymo Research) and stored at −80°C. Preserved specimens were transferred to the One Health Genomic Laboratory at the Universidad Nacional Sede Medellín and shipped to the University of Wisconsin-Madison for DNA extraction and sequencing. In addition to fecal specimens, serum samples and nasal swabs were collected for SARS-CoV-2 testing. Recent infections were assessed using the Abbott Architect SARS-CoV-2 IgG antibody assay in serum (Abbott Park, IL). Active infections were determined through genomic DNA extraction from nasal swabs using Gene E RT-PCR, as previously described (40).

### DNA extraction

DNA extraction from fecal specimens was conducted using the QIAamp PowerFecal Pro DNA kit (Qiagen). The extraction process followed the manufacturer’s protocol, with a modification at step 1, where the input material consisted of 50 μL of fecal slurry in DNA/RNA Shield, 500 μL of CD1 buffer, and 300 μL of ATL buffer (Qiagen; not included in the kit). Bead beating was performed using a TissueLyser II (Qiagen) for two cycles, each lasting 5 min at 25 Hz. Between cycles, adaptors containing the specimens were repositioned so that samples that were closer to the machine body were further away in the second cycle. Finally, samples were eluted in a final volume of 50 μL RNase-free water and stored at −80°C.

### 16S rRNA metabarcoding sequencing and analysis

Qiagen Genomic Services conducted 16S rRNA microbiome profiling using the QIAseq 16S/ITS Screening Panel for library preparation. First, starting with 1 ng of DNA, target regions were selected and amplified through 12 cycles of PCR. Samples underwent cleanup using QIAseq Beads (Qiagen), followed by the addition of sequencing adapters and enrichment in a second PCR of 12 cycles. After a second bead cleanup, the libraries underwent quality control assessment using capillary electrophoresis (Tape D1000). High-quality libraries were then pooled in equimolar concentrations, determined by the Bioanalyzer automated electrophoresis system (Agilent Technologies). The library pools were quantified using qPCR, and the optimal concentration was used to generate clusters on the surface of a flow cell before sequencing on a MiSeq (Illumina Inc.) instrument (2 × 276).

Raw V4–V5 16S rRNA fragment reads were processed using a QIIME2 pipeline (41). The DADA2 plugin (42) was utilized for trimming reads, removing sequences with ambiguous nucleotides and chimeras, and discarding singletons. The remaining sequences, with a length of approximately 370 bp, were clustered into operational taxonomic units (OTUs) at a 99% identity level. We based our decision to use 99% identity level OTUs on the fact that human gut microbiota taxa are well-defined and well-documented in the reference databases compared to what can be expected of a non-explored environment, where undescribed taxa can be more properly described with an amplicon variant sequence approach.

Taxonomic classification was performed using the q2-feature-classifier plugin with the Bayes machine-learning classifier method (43) trained with the Greengenes 515F/806R database v.13.8 (44) with OTUs identified as Mitochondria, Chloroplast, or Archaea discarded. After removing low-abundance OTUs (≤0.01% total sampling) and samples (1,000 sequences), two individuals from Km11 were discarded, resulting in a final sample size of 98 individuals.

Alpha and beta diversity analyses were conducted in R in the phyloseq package (45). To assess the overall influence of donor location, a permutational multivariate analysis of variance (PERMANOVA) with 999 permutations was performed on weighted UniFrac distances using the vegan package (46). To identify microbial composition differences between the locations, two approaches were employed. (i) Identify OTUs with differential abundance using the Wald significance test implemented in DESeq2 (47), where a geometric mean is calculated for each OTU across all samples, with the OTU counts divided by this mean, and the median of these ratios being the size factor for a sample. Later, a hypothesis testing is performed with Km11 as the treatment group and Leticia as the control (threshold cutoff, *α* = 0.01). (ii) Evaluate the differential abundance of selected BloSSUM and VANISH taxa by using the Wilcoxon rank-sum test. Per sample, we add all the reads from the selected taxa, calculate the relative abundance, and compare statistically this result between the two locations. For BloSSUM taxa, *Bacteroidaceae*, *Verrucomicrobiaceae*, and *Rikenellaceae* were chosen. For the VANISH taxa, *Prevotellaceae* plus *Paraprevotellaceae* (referred to as *Prevotellaceae*), *Succinivibrionaceae*, *Spirochaetaceae*, and the clostridiales *Lachnospiraceae* with *Ruminococcaceae* as one group (referred to as *Lachnospiraceae*) were chosen. For microbiota functional analysis, PICRUSt 2.0 (48) was utilized to predict biological pathways. Results were classified with the MetaCyc database (49) and differential abundances between Km11 and Leticia microbial pathways were evaluated using a Wald significance test within the ggpicrust2 package (50).

To describe the Leticia bacterial microbiota structure in a regional context, we performed a comparative analysis of our data set against previously reported Amazonian and non-Amazonian Colombian microbiotas. We included data sets that met two criteria: use of the V4 region of the 16S rRNA sequence and reads with >100 bp in length. The final analysis included nine data sets, divided into six Amazonian populations: (i) the urban and rural Leticia sampling as one group; (ii) the urban Belém and rural indigenous (iii) Suruí, (iv) Tupaiú, and (v) Xikrin (4) communities in Brazil; and the (vi) rural indigenous Tsimané community in Bolivia (34); and three non-Amazonian data sets, two urban Colombian (vii) Bogotá and (viii) Medellín) (45); and (ix) an urban American cohort from Ohio, subsampled from the American Gut Project (46) (Table S1). Given that some data sets only sampled adult donors, infant samples (<15 years old) were removed before the analysis. Raw 16S rRNA reads were trimmed to 130 bp and preprocessed using the QIIME2 pipeline. Comparative analyses of alpha and beta diversity and selected BloSSUM and VANISH taxon abundance were performed in R.

### 18S rRNA metabarcoding sequencing and analysis

For the same DNA samples used for 16S rRNA analysis, we carried out eukaryotic analysis using the VESPA (Vertebrate Eukaryotic Endo-Symbiont and Parasite Analysis) metabarcoding protocol (51) targeting the 18S rRNA gene V4 region. Library pools were sequenced using MiSeq (Illumina Inc.) instrument with a 300 × 300 cycle chemistry. Raw reads were processed using a QIIME2 pipeline with OTUs at a 99% identity level classified using the PR2 reference sequence database v.5.0 (52). OTUs with unassigned or incomplete taxonomy were manually classified using the full NCBI nucleotide database. Three Km11 samples with ≤1,000 sequences were discarded from the final data set. PERMANOVA tests were used to evaluate the influence of parasitic protists and nematodes in bacterial community structure.

## RESULTS

We evaluate the impact of urbanization on local microbiota structure in the gut microbiota of Leticia by comparing the peri-urban rural indigenous community of Kilometro 11 (Km11) with the non-indigenous urban population (Leticia). Participants completed surveys that provided insights into the conditions of the community (Table 1). The cohort showed a representative sex distribution (Leticia, 60% female, 40% male; Km11, 58% female, 42% male) with donors ranging from 5 to 82 years (Leticia, median age 36.5 years old; Km11 38.0 years old). As expected, ethnic identification varied significantly by location, with 95% of the Km11 community identifying as indigenous (primarily Wuitoto, Ticuna, and Murui). In contrast, only one donor from Leticia identified as indigenous, with most self-describing as *mestizos* (mixed-race). Most Km11 participants typically spend the day around their residence with high contact with livestock (82%), raising and sacrificing mostly poultry (chickens and ducks). Conversely, companion animal contact showed no significant difference between sites. Finally, over 90% of the donors stored water at home for domestic use.

**TABLE 1:** Socioeconomic and health parameters for Leticia and Km11 groups^*a*^

Socioeconomic and health parameters	Permanova result	Parameter levels	Leticia (%)	Km 11 (%)
Sex	*F* = 0.56, *R*^2^ = 0.01, *P* = 0.62	Female	60	59
		Male	40	41
Age group	*F* = 2.03, *R*^2^ = 0.04, *P* = 0.08	1–15 yo	5	10
		16–30 yo	25	22
		31–85 yo	70	68
Ethnic identification	*F* = 10.18, *R*^2^ = 0.10, *P* = 1.0E−3	Indigenous	5	91
		Non-indigenous	95	9
Scholarity level	*F* = 1.97, *R*^2^ = 0.10, *P* = 0.02	None	0	3
		Elementary school	20	27
		High school	25	60
		Technical school	40	6
		Undergraduate education	10	3
		Graduate education	5	1
Occupation	*F* = 1.12, *R*^2^ = 0.05, *P* = 0.33	Unemployed	0	17
		Employee	65	14
		Independent	15	13
		Housewife	10	38
		Student	10	18
Housing social strata^*b*^	*F* = 1.58, *R*^2^ = 0.05, *P* = 0.14	Rural	0	76
		Level 1	20	21
		Level 2	50	4
		Level 3	30	0
Number of house inhabitants	*F* = 0.90, *R*^2^ = 0.02, *P* = 0.45	1	10	5
		2–4	35	28
		5 or more	55	67
Place where stay most of the day	*F* = 0.86, *R*^2^ = 0.02, *P* = 0.52	Home	30	78
		Work/Chagra	70	21
		School	0	1
Livestock contact^*c*^	*F* = 8.01, *R*^2^ = 0.08, *P* = 1.0E−3	Yes	5	82
		No	95	18
Companion animal contact	*F* = 1.29, *R*^2^ = 0.01, *P* = 0.21	Yes	55	22
		No	45	78
Insecticide use	*F* = 1.43, *R*^2^ = 0.08, *P* = 0.19	Yes	95	73
		No	5	27
Mosquito net use	*F* = 6.44, *R*^2^ = 0.06, *P* = 2.0E−3	Yes	25	94
		No	75	6
Water storing at home	*F* = 1.01, *R*^2^ = 0.01, *P* = 0.35	Yes	90	94
		No	10	6
Healthcare coverage	*F* = 3.37, *R*^2^ = 0.06, *P* = 0.01	No coverage	5	3
		Subsidized coverage	15	86
		Private coverage	80	12
Hospitalization in the last year	*F* = 0.39, *R*^2^ = 4.6E−3, *P* = 0.79	Yes	0	12
		No	100	88
Chronic diseases history	*F* = 1.49, *R*^2^ = 0.01, *P* = 0.18	Yes	35	22
		No	65	78
Vector-borne diseases history	*F* = 1.12, *R*^2^ = 0.01, *P* = 0.28	Yes	25	18
		No	75	82
Chemiluminescence assay IgG SARS-CoV-2^*d*^	*F* = 1.06, *R*^2^ = 0.02, *P* = 0.32	Yes	55	58
		No	45	38
		Not measured	0	4
Gen E RT-PCR SARS-CoV-2	*F* = 0.81, *R*^2^ = 8.4E−3, *P* = 0.42	Yes	5	9
		No	95	91
Medication use in the last month	*F* = 2.39, *R*^2^ = 0.02, *P* = 0.08	Yes	70	45
		No	30	55
Smoking history	*F* = 1.94, *R*^2^ = 0.02, *P* = 0.14	Yes	5	6
		No	95	94

^*a*^ PERMANOVA results for each parameter with *P* value indicating statically significant influence on microbiota structure.

^*b*^ Housing social strata based in the Colombian official strata divisions, rural and urban one to six levels.

^*c*^ Livestock contact includes raising and/or sacrificing domestic animals.

^*d*^ Three samples from Km11 were excluded because of the lack of biological material for testing.

Both groups reported low frequencies of historical diagnoses of vector-borne and chronic diseases (18–35%, with cardiovascular disease and hypertension being the most prevalent). Over 50% of donors at both locations had recent SARS-CoV-2 infection indicated by an IgG antibody assay, and less than 10% had an active positive infection based on RT-PCR (Table 1) although they were asymptomatic at the time of sampling. Medication use reported during the preceding month was high, particularly in Km11, especially for analgesics like paracetamol and ibuprofen.

**Fig 1:**
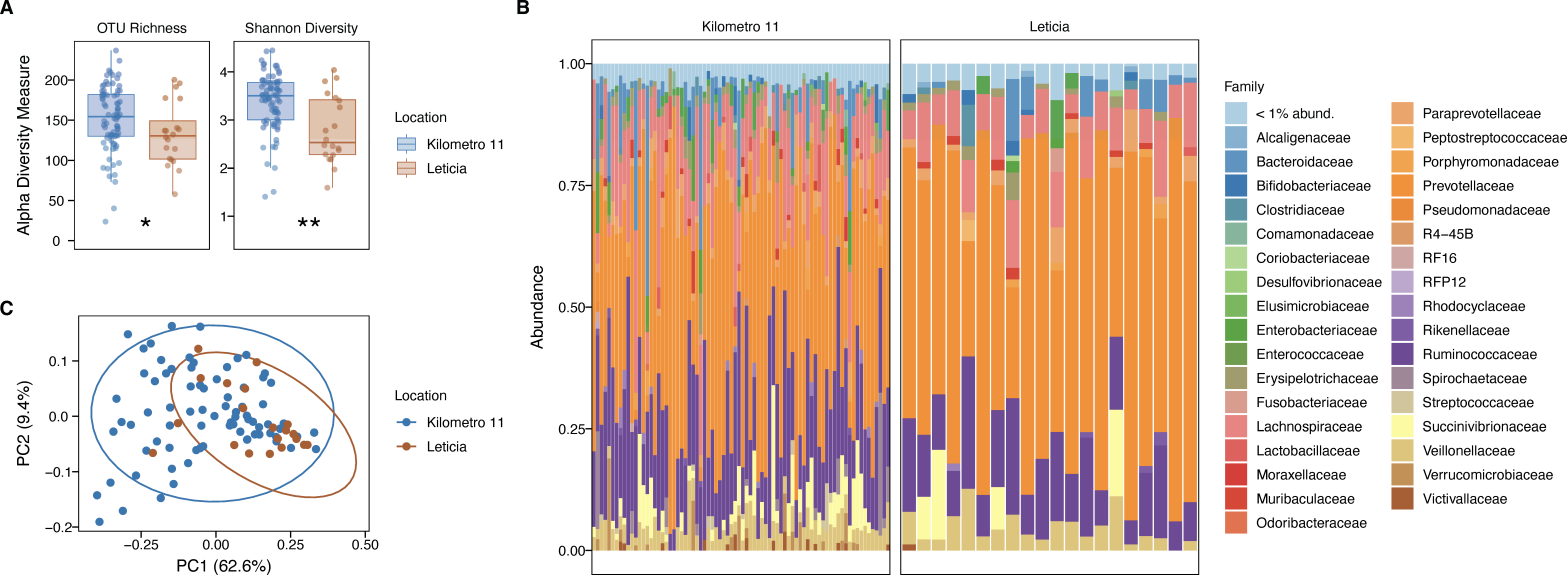
Leticia gut bacteria microbiota alpha and beta diversity analysis. (**A**) Alpha diversity estimators, number of OTUs (richness) and Shannon Index (diversity). Difference between locations evaluated using the Wilcoxon rank-sum test. ns: *P* > 0.05, *: *P* ≤ 0.05, **: *P* ≤ 0.01, ***: *P* ≤ 0.001, and ****: *P* ≤ 0.0001. (**B**) Relative bacterial family abundance for locations. Families with <1% abundance were merged into one group. (**C**) PCA plot of bacterial community structure based on the weighted UniFrac distances.

To describe the bacterial microbiota compositions of the two groups, we sequenced the V4–V5 16S rRNA region. After preprocessing the reads, our final metabarcoding data set comprised an average of 28,798 ± 15,564 sequences per sample, distributed across 466 OTUs at 99% resolution level (Table S1). Most samples exhibited good taxonomic coverage, reaching the maximum number of OTUs with a subsampling of 10,000 sequences (Fig. S1A). Both richness (number of OTUs, *P* value = 0.045) and diversity (Shannon index, *P* value = 0.001) were significantly reduced in the Leticia group even after rarefaction (Fig. 1A; Fig. S1B). At the family level, *Prevotellaceae* was the most abundant group in the sampling, with >38% of the overall abundance for both locations (Fig. 1B), followed by *Ruminococcaceae* (~15%). We evaluated the influence of the surveyed variables on microbiota structure using a PERMANOVA analysis. The results revealed a statistically significant influence of location (*F* = 7.904, *R*² = 0.08; *P* = 0.002), with the Leticia samples clustering in the PCA plot (Fig. 1C). Other variables also differed between locations, such as ethnic identification, livestock contact, mosquito net use, healthcare coverage, and educational level, and these were significantly associated with the gut microbiota community structure (Table 1).

**Fig 2:**
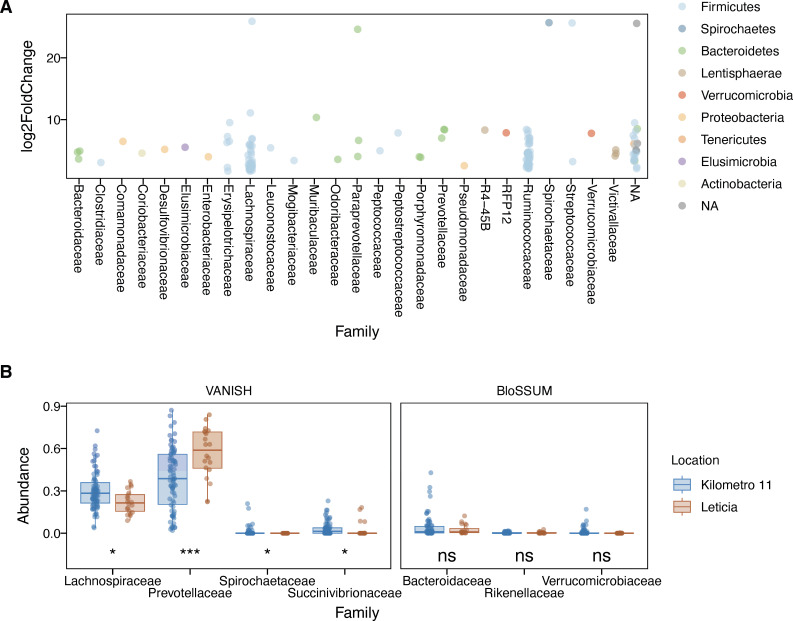
Differential abundance analysis between Leticia and Km11. (**A**) Significant OTUs according to the Wald significance test. Km11 as the treatment and Leticia as the control group, with an α = 0.01 threshold cutoff. (**B**) Differential abundance of VANISH and BloSSUM taxa. Difference between locations evaluated using Wilcoxon rank-sum test. ns: *P* > 0.05, *: *P* ≤ 0.05, **: *P* ≤ 0.01, ***: *P* ≤ 0.001, and ****: *P* ≤ 0.0001.

Next, to identify the taxa driving differences between Leticia and Km11, we analyzed bacterial differential abundance using two approaches. For the Wald significance test, 135 out of 466 total OTUs showed a statistically significant log fold change (Fig. 2A; Table S2), all of them with an increased abundance in Km11 compared to Leticia. Most of these OTUs (55%) belong to the Clostridial families *Ruminococcaceae* and *Lachnospiraceae*, which were the most represented groups in these increased OTU list (55%) (Table S2). The selected family abundance analysis (Fig. 2B; Table S3) showed significant reductions of VANISH taxa in Leticia, with *Spirochaetaceae* locally extinct while *Prevotellaceae* increased in the urban setting leading to a homogenization of the microbial structure. No significant difference was observed for BloSSUM groups, being in low abundance for most donors.

**Fig 3:**
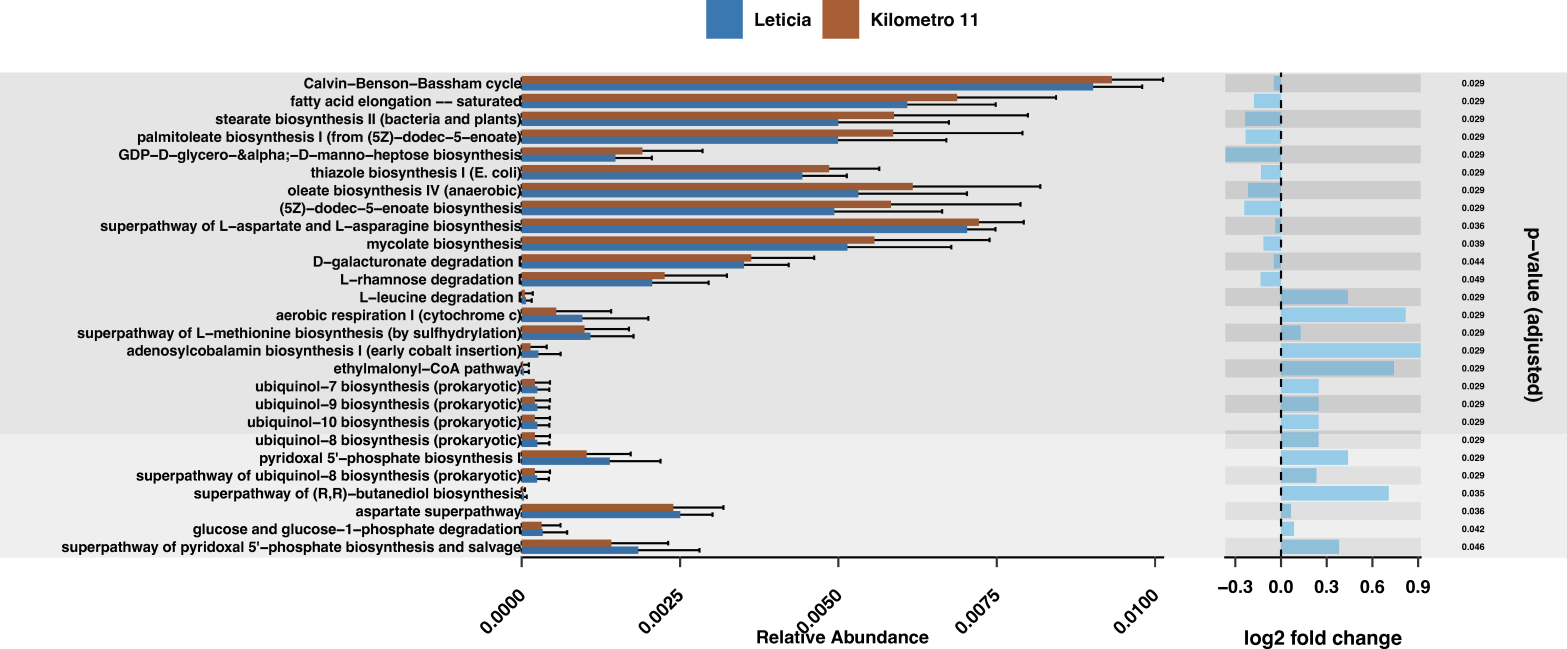
Predictive bacterial metabolic pathways analysis. (Left) Relative abundance of pathways with differential abundance between locations. (Right) Logarithmic fold change between pathways based on the Wald significance test. Km11 as the treatment and Leticia as the control group, with an α = 0.01 threshold cutoff.

In order to infer physiological implications of these bacterial microbial repertoires, we performed a predictive functional analysis finding a total of 331 predicted metabolic pathways. Using a Wald significance test, we identified 30 pathways with significant differences in relative abundance between the two locations (Fig. 3; Table S4). Compared to Leticia, 14 pathways were increased in Km11, with half of these belonging to fatty acid and lipid biosynthesis pathways. A total of 16 pathways were increased in Leticia, including pathways associated with aerobic respiration like cofactors biosynthesis pathways.

**Fig 4:**
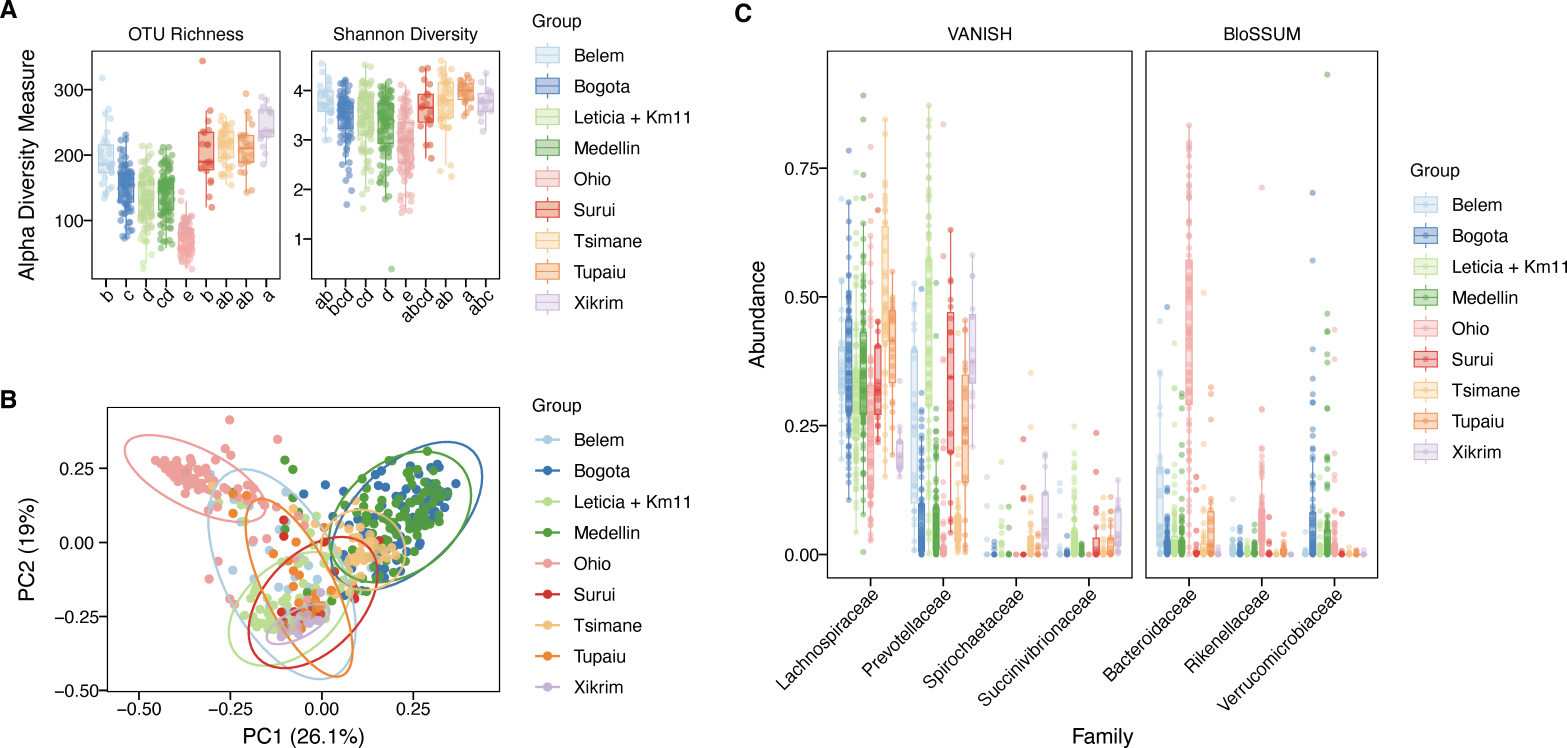
Amazonian and non-Amazonian gut bacteria microbiota data sets alpha and beta diversity comparative analysis. (**A**) Alpha diversity estimators of number of OTUs (richness) and Shannon Index (diversity). Letters reflect grouping and differences between data sets evaluated using the Tukey HSD test. (**B**) PCA plot of bacterial community structure based on the weighted UniFrac distances. (**C**) Abundance of VANISH and BloSSUM taxa across bacteria microbiota data sets.

To evaluate the bacterial community compositions in a regional context, we performed a comparative gut bacteria microbiota analysis comprising nine datasets, including six Amazonian data sets (two urban groups including our sampling, and four rural groups) and three urban non-Amazonian data sets, for a total of 489 samples (File S1; Fig. S2). For the alpha diversity (Fig. 4A), rural Amazonian groups showed higher richness than urban communities, where Leticia has similar values to the other Colombian urban microbiotas. We evaluated the effect of the differential sampling effort between data sets (Fig. S3A) using a rarified data set, finding that read counting did not affect the interpretation of the richness analysis (Fig. S3B). We performed a PERMANOVA analysis to evaluate how much the sampled location can explain the microbiota structure (*F* = 37.94, *R*² = 38.09, *P* = 0.001). This was visualized in the PCA, where Ohio samples formed a separated cluster from three South American subgroups, Colombian urban Medellín-Bogotá, one transitional group for the Amazonian rural Tsimané, and the Leticia samples indistinct from Brazilian rural and urban populations (Fig. 4B). The clustering of Leticia and Brazilian samples is explained in the selected family analysis (Fig. 4C; File S1), where VANISH taxa *Prevotellaceae* and *Succinivibrionaceae* are increased compared to the other populations, while BloSSUM groups dominated in the urban populations (*Bacteroidaceae* and *Rikenellaceae* in Ohio, and *Verrucomicrobiaceae* in Bogotá and Medellín) are reduced. Belém, the other Amazonian city evaluated, is distinguished from Leticia for the higher abundance of *Bacteroidaceae,* even with some samples clustering with the Ohio samples.

**Fig 5:**
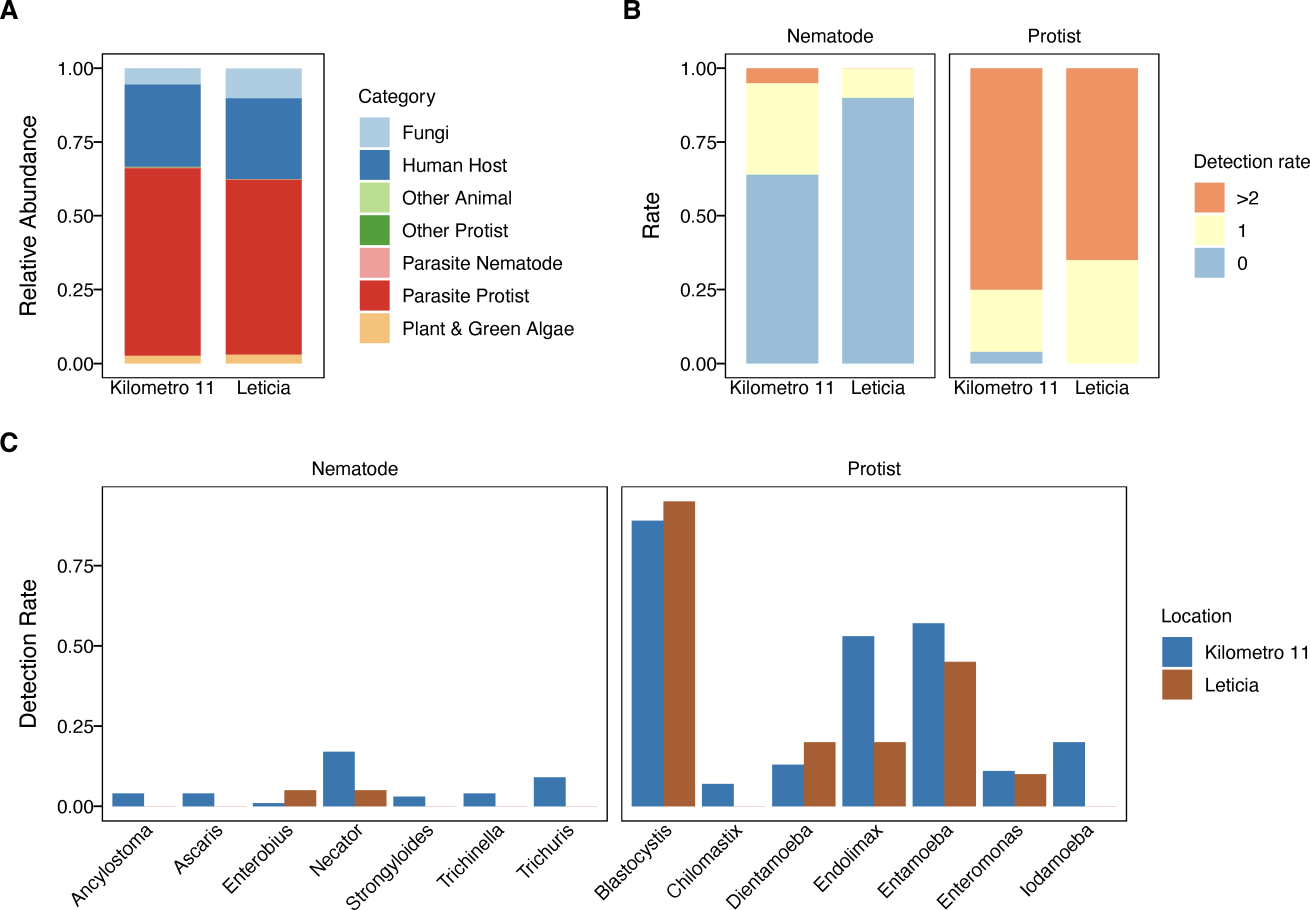
Leticia eukaryotic parasite metabarcoding description. (**A**) Relative sequence abundance from the 18S rRNA metabarcoding for each taxonomic category. (**B**) Overall detection rate of parasite nematodes and protists in Km11 and Leticia samples, and (**C**) breakdown of taxa detected.

To describe the parasitic protists and nematodes associated with the Leticia gut microbiota, we performed an 18s rRNA analysis using the VESPA (Vertebrate Eukaryotic endoSymbiont and Parasite Analysis) protocol for eukaryotic endosymbiont metabarcoding (51). Amplicons were generated targeting the 18S rRNA gene V4 region, sequenced, and recovered reads were classified into seven taxonomic categories using the PR2 reference sequence database (Human Host, Parasite Protist, Parasite Nematode, Other Animal, Other Protist, Fungi, and Plant & Green Algae) (File S2). Parasitic protist sequences were the most common group, followed by the human host. Parasitic nematodes were recovered in a low abundance (<1%) (Fig. 5A; File S2). Most individuals in both locations were infected with two or more parasitic protist taxa (Fig. 5B). Nematode infections were mostly detected in rural Km11 (Fig. 5B). Only two nematode taxa, *Enterobius* and *Necator*, were detected in urban Leticia, while seven genera were found in Km11 at rates ranging from 1% to 17% (Fig. 5C). Five protist taxa were detected in both locations, with *Blastocytis* being the most common, detected in >85% of samples from both locations (Fig. 5C).

**Fig 6:**
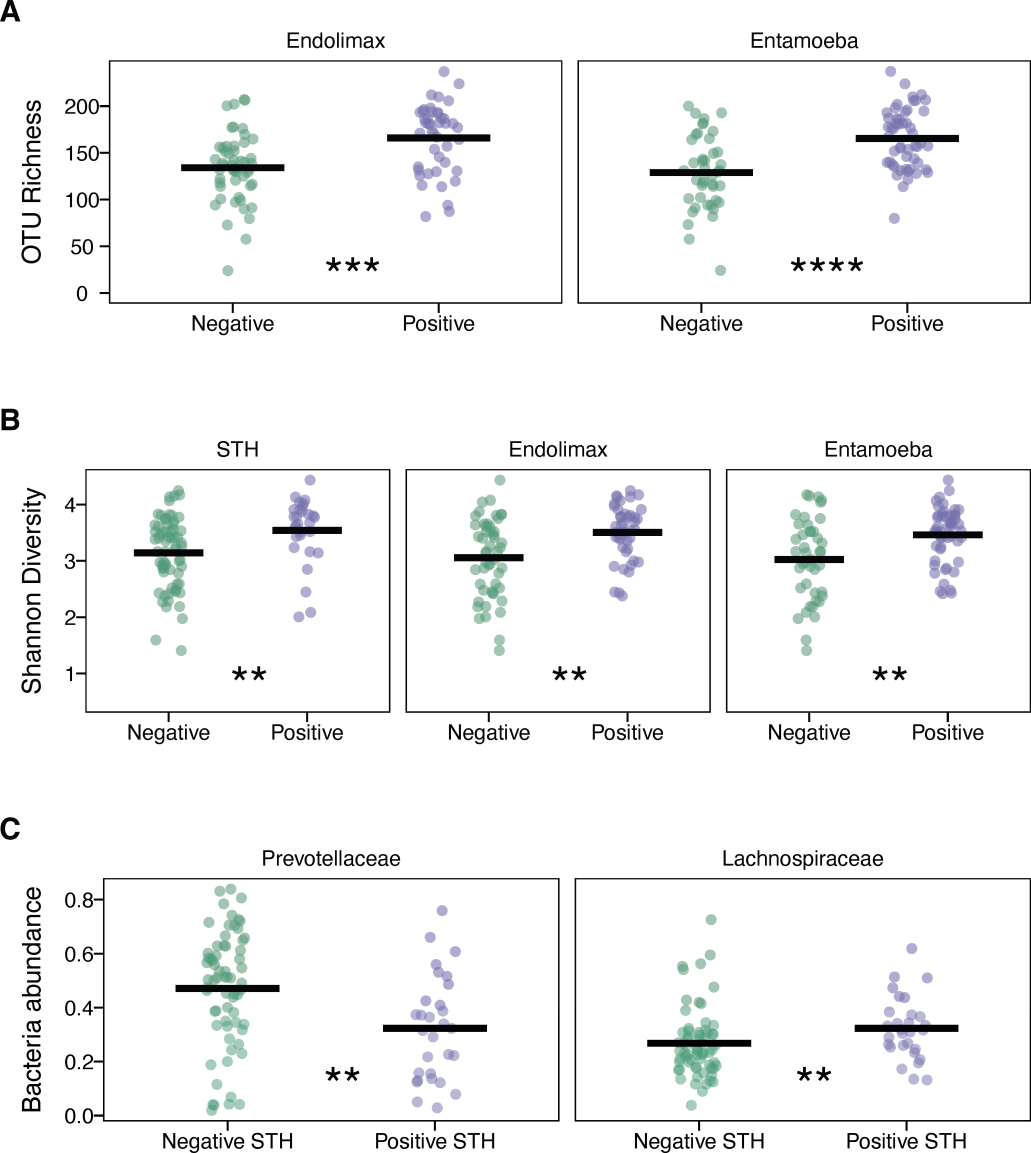
Differential analysis between parasite positive and negative Leticia (both urban and rural) samples. (**A**) Richness (observed number of OTUs) for *Endolimax* and *Entamoeba* infection. (**B**) Diversity (Shannon index) for STH, *Endolimax*, and *Entamoeba* infection. (**C**) *Prevotellaceae* and *Lachnospiraceae* abundance for STH infection. Difference between infection status evaluated using the Wilcoxon rank-sum test. ns: *P* > 0.05, *: *P* ≤ 0.05, **: *P* ≤ 0.01, ***: *P* ≤ 0.001, and ****: *P* ≤ 0.0001.

Given the extensive literature indicating that parasite infection influences the host microbial environment, we tested whether there was an association with protist or helminth infection and either bacterial diversity or abundance. PERMANOVA tests were used to evaluate alpha diversity metrics for the number of observed OTUs (richness), Shannon index (diversity), and abundance of *Prevotellaceae* and *Lachnospiraceae* family of bacteria. The Leticia and Km11 data sets were combined into one group, and comparisons were made between positive and negative infection status with any STH taxa or infection with any of the five most prevalent protists (*Blastocystis*, *Dientamoeba*, *Endolimax*, *Entamoeba*, and *Enteromonas*). Parasite infection was significantly associated with increased richness of bacterial tax for *Endolimax* (*F* = 12.137, *R*² = 0.115, *P* = 0.001) and *Entamoeba* (*F* = 19.834, *R*² = 0.176, *P* = 0.001). Infection with the following protist and nematode parasites was associated with increased diversity: *Endolimax* (*F* = 12.618, *R*² = 0.119, *P* = 0.001), *Entamoeba* (*F* = 12.625, *R*² = 0.119, *P* = 0.003), and STH infection (*F* = 6.919, *R*² = 0.069, *P* = 0.007). For the bacterial taxonomic abundance, STH infection influenced both *Prevotellaceae* (*F* = 6.722, *R*² = 0.068, *P* = 0.001) and *Lachnospiraceae* (*F* = 4.753, *R*² = 0.048, *P* = 0.018). All results were confirmed using the Wilcoxon rank-sum test comparing positive and negative infection samples (Fig. 6).

## DISCUSSION

This study sheds new light on the gut bacterial microbiota of urban and rural populations in the Colombian Amazon. In urban Leticia, we found lower bacterial diversity than in Km11 communities, reflecting reduced abundance of families related to non-processed foods (VANISH taxa), such as *Lachnospiraceae*, *Ruminococcaceae*, *Succinivibrio*, and *Spirochaetaceae*. The latter includes spirochetes of the genus *Treponema*, which are strongly associated with traditional rural populations of non-“Western” lifestyles (35). Similar reductions in microbiota diversity have also been reported in other locations undergoing demographic and cultural transitions (53, 54). However, a noteworthy difference for Leticia is the increase of *Prevotella* instead of taxa associated with processed foods diets (BloSSUM taxa) in the urban setting.

Decreased microbial diversity in samples from the urban, mostly non-indigenous Leticia population compared to the rural, mostly indigenous Km11 population may be explained by lifestyle differences. Most of the Km11 donors practice activities such as small poultry farming for economic sustenance (55). Outdoor activity (56) and livestock raising (57, 58) have been shown to enrich microbiota diversity. Also, the diet in urban Leticia has changed significantly in recent decades compared to rural areas of the Amazon, with reduced consumption of traditional non-industrialized foods like fish broth, wild animals, cassava-derived products like casabe and farinha, and fruit-based juices, and increased consumption of products such as packaged chicken, eggs, rice, canned foods, and powdered drink mixes of coffee, cocoa, or fruit (55).

For the comparative analyses, our initial assessment was to use the PCA to look for batch effects, a common problem for microbial studies (59). The analysis did not show any clustering based on the original sample study. The gut microbiota Leticia population was similar to that described within the Brazilian Amazonian urban and rural communities (4, 6), with a high *Prevotellaceae* abundance and in general low presence of BloSSUM taxa. This pattern may be explained by similarities in the diet of these riverine communities along the Amazonian basin, which is still mostly composed of fish and polysaccharide-rich foods like cassava (4). This differs from the lowland forest Tsimané community in Bolivia (34), the other Amazonian group evaluated, which has a diet rich in plant foraging and wild animals (60). The taxonomic differences with Bogotá and Medellín, the other Colombian urban microbiotas, where BloSSUM taxa are more abundant, seem to reinforce the concept of a “tropical urban” category to describe microbiota of habitats of urban areas in tropical regions that are in different stages of microbiota “westernization” compared to non-tropical populations (4), although the different levels of urban transition, exemplified in the taxonomic differences between Leticia and Belém, indicate the necessity to further sample more Amazonian urban locations.

We also described the Leticia gut eukaryotic parasite community using a recently developed 18S rRNA metabarcoding protocol, VESPA (51). This method has been proved to be useful to describe endosymbiotic communities, with a higher rate of positive parasite infection description compared to traditional microscopy methods. For our sampling, we found a high abundance of intestinal parasites in both urban and rural samples, with *Endolimax* and *Entamoeba* presence having a positive effect on microbial richness and diversity. Protist infection is already known to influence microbiota structure, being associated with higher microbial richness and abundance of VANISH taxa like *Prevotellaceae* and *Ruminococcaceae* (12, 13). We found that STH parasites are associated with higher bacterial microbial diversity and VANISH taxa abundance. A similar result, increased bacterial species diversity and *Prevotellaceae* abundance*,* was reported in a cohort of Colombians harboring mixed STH infections (61). While VESPA has been shown to be more sensitive than microscopy in detecting the *presence* of helminth and protozoan taxa in human and non-human fecal samples, we acknowledge that quantification of *intensity* of infection using sequencing-based approaches can be limited by inefficiencies in DNA extraction from parasite eggs. Nevertheless, these results indicate potential parasite-microbiome interactions that could influence human health. This phenomenon has been extensively studied for both protists and helminths, which have been proposed to be beneficial by protecting against allergic and metabolic diseases (62–64).

Finally, predictive metabolic analysis indicates several significant results that may be relevant to the health outcomes of the microbiota structure in the study area. Urban samples had relative depletion of fatty acid biosynthesis pathways. This finding may be explained by the reduced abundance of *Lachnospiraceae*, which are key producers of butyrate and other short-chain fatty acids and a decrease in diets high in saturated fatty acids (65). Interestingly, depletion in short-chain fatty acid producers was also observed in *Trichuris*-infected individuals in a large study spanning Côte d’Ivoire, Laos, and Tanzania (66). There was also an increase in the aerobic respiration pathways in urban samples, which may indicate increased saturated fatty acid consumption. These changes can increase the epithelial oxygenation in the colon, triggering a microbiome structure shift characterized by an elevated abundance of facultatively aerobic bacteria compared to the healthy microbiota composition dominated by anaerobic bacteria (67). Finally, although *Prevotellaceae* has been associated with a healthy microbiota, the high prevalence found in Leticia should be carefully interpreted, as this group has also been linked with inflammatory autoimmune diseases like rheumatoid arthritis (68).

Differences in the taxonomic composition and predicted physiology of the urban and rural Leticia microbiota documented in this study are likely associated with cultural and health transitions and intestinal protozoa and STH infections. These findings have relevance to public health, as such changes may underlie increases in chronic non-communicable diseases in the region, highlighting the need for further investigations into microbiota dynamics among urban and rural populations across the Colombian Amazon. This expanded analysis will be important for enhancing our understanding of the local health transitions and implementing proactive measures to improve public health outcomes and healthcare system preparedness.

## Data Availability

Sequencing data were deposited in the NCBI SRA database (bioproject accession number PRJNA1246579).

## SUPPLEMENTAL MATERIAL

File S1 msphere.00788-25-s0001.xlsx Supplemental information regarding gut bacterial microbiota analysis of Amazonian and non-Amazonian data sets.

File S2 msphere.00788-25-s0002.xlsx Supplemental information regarding eukaryotic microbiota analysis o f Amazonian and non-Amazonian datasets

Figure S1 msphere.00788-25-s0003.tiff Complementary Leticia gut bacteria microbiota alpha diversity analysis.

Figure S2 msphere.00788-25-s0004.tiff Relative bacterial family abundance for Amazonian and non-Amazonian data sets.

Figure S3 msphere.00788-25-s0005.tiff Complementary gut bacteria microbiota alpha diversity analysis in Amazonian and non-Amazonian data sets.

Legends msphere.00788-25-s0006.docx Supplemental material legends.

Table S1 msphere.00788-25-s0007.xlsx OTU count table for 16S rRNA Leticia sampling.

Table S2 msphere.00788-25-s0008.xlsx Wald significance test for OTU abundance between Leticia and Km11 locations.

Table S3 msphere.00788-25-s0009.xlsx Relative abundance of BloSSUM and VANISH taxa per sample for Leticia sampling.

Table S4 msphere.00788-25-s0010.xlsx Metacyc database classification of the metabolic pathways with a differential abundance between Leticia and Km11 based on the Wald significance test.

ASM does not own the copyrights to Supplemental Material that may be linked to, or accessed through, an article. The authors have granted ASM a non-exclusive, world-wide license to publish the Supplemental Material files. Please contact the corresponding author directly for reuse.
