# Supplementary figures and images for: Gut microbiota and parasite dynamics in an Amazonian community undergoing urbanization in Colombia
Source: mSphere. 2026 Jan 28;11(2):e00788-25. doi: 10.1128/msphere.00788-25 (PMC12931277; doi:10.1128/msphere.00788-25)

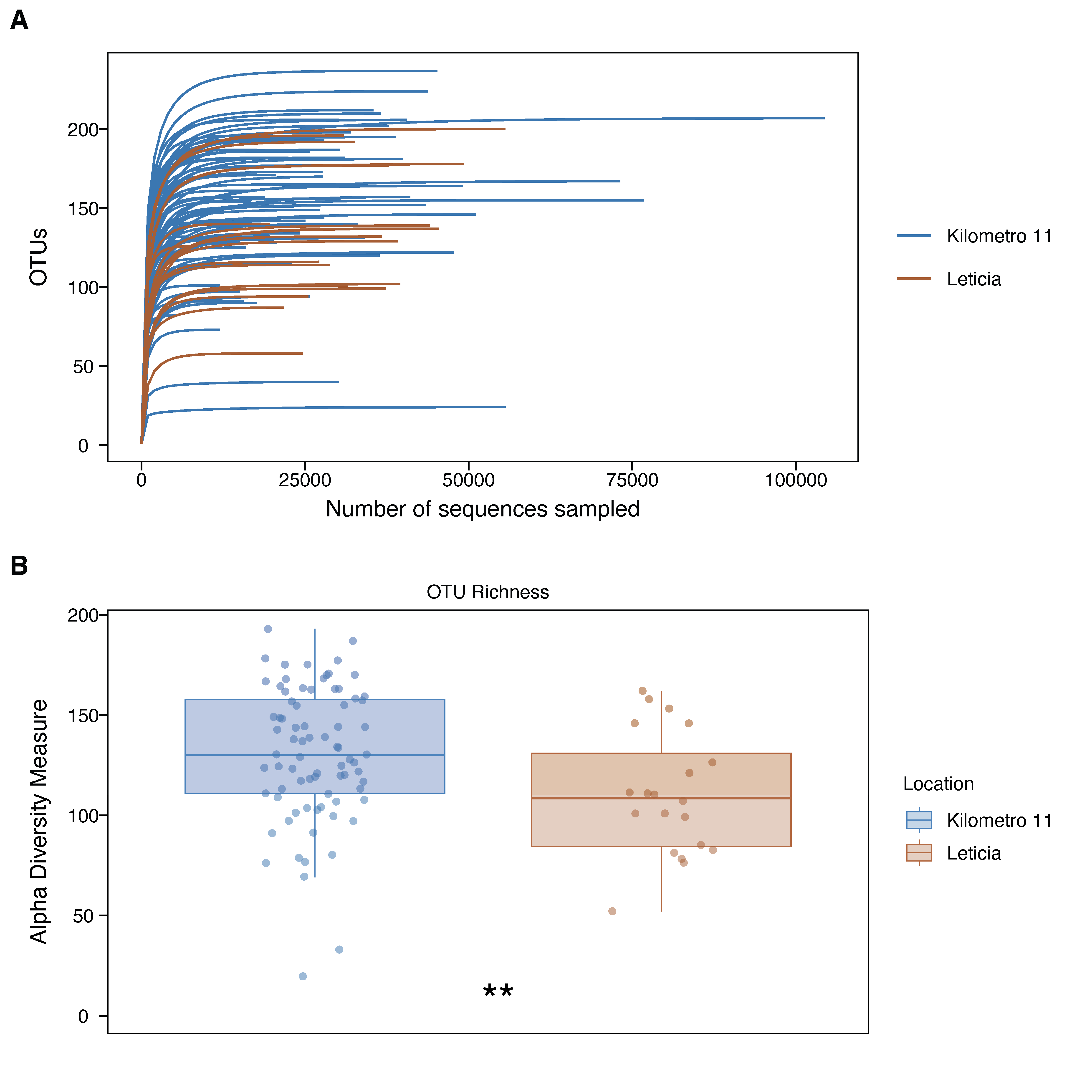

Supplement: Figure S1 — Complementary Leticia gut bacteria microbiota alpha diversity analysis. [file msphere.00788-25-s0003.tiff]

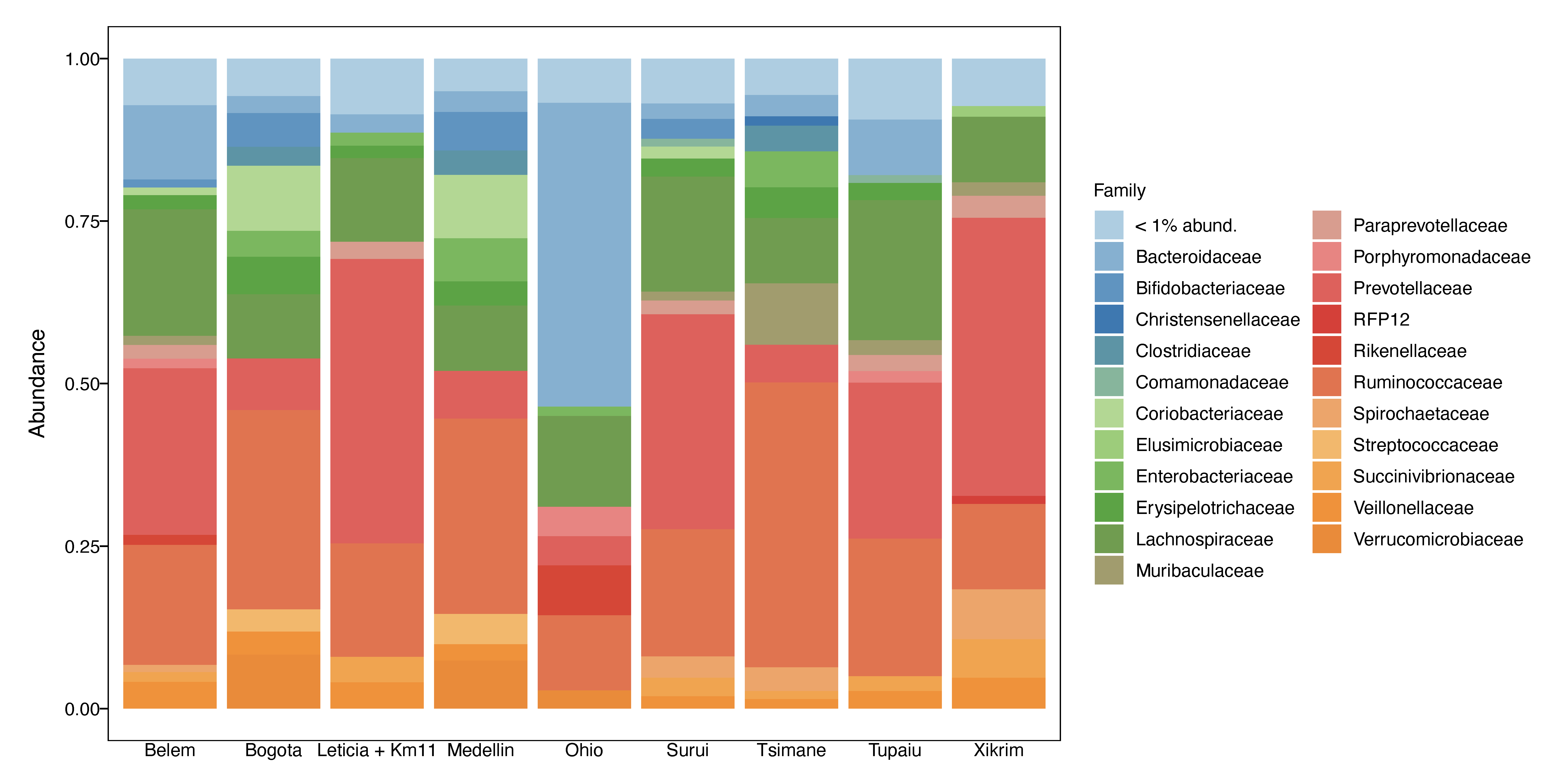

Supplement: Figure S2 — Relative bacterial family abundance for Amazonian and non-Amazonian data sets. [file msphere.00788-25-s0004.tiff]

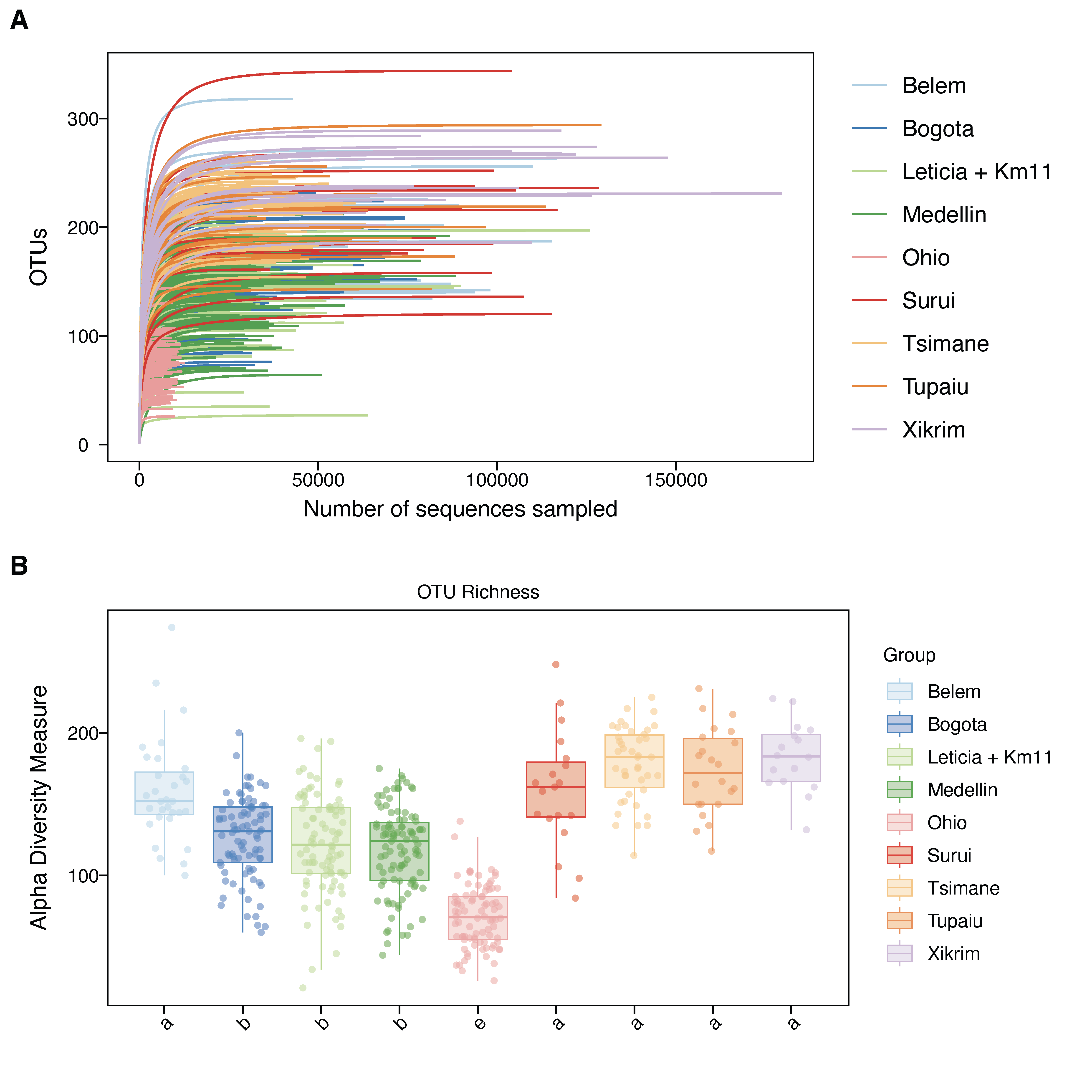

Supplement: Figure S3 — Complementary gut bacteria microbiota alpha diversity analysis in Amazonian and non-Amazonian data sets. [file msphere.00788-25-s0005.tiff]
