# Supplementary material for: Gut microbiota and parasite dynamics in an Amazonian community undergoing urbanization in Colombia
Source: mSphere. 2026 Jan 28;11(2):e00788-25. doi: 10.1128/msphere.00788-25 (PMC12931277; doi:10.1128/msphere.00788-25)
Supplement: Legends — Supplemental material legends. [file msphere.00788-25-s0006.docx]

**Supplementary Material Legends**

**Table S1.** OTU count table for 16S rRNA Leticia sampling. Each OTU with taxonomic description, Phylum and Family.

**Table S2.** Wald Significance Test for OTUs abundance between Leticia and Km11 locations. Abundance differences expressed in log fold change, with statistical significance indicated with the FDR-adjusted p-value using the Benjamini-Hochberg multiple-inference correction.

**Table S3.** Relative abundance of BloSSUM and VANISH taxa per sample for Leticia sampling.

**Table S4**. Metacyc database classification of the metabolic pathways with a differential abundance between Leticia and Km11 based on the Wald Significance Test.

**File S1.** Supplemental information regarding gut bacterial microbiota analysis of Amazonian and non-Amazonian datasets. **Sheet 1.** Sample list for datasets used for the comparative microbiota analysis. **Sheet 2.** OTU count Table for 16S rRNA Amazonian and non-Amazonian sampling. Each OTU with taxonomic description, Phylum and Family. **Sheet 3.** Relative abundance of BloSSUM and VANISH taxa per sample for Amazonian and non-Amazonian.

**File S2.** Supplemental information regarding eukaryotic microbiota analysis of Amazonian and non-Amazonian datasets. **Sheet 1.** OTU count Table for 18S rRNA Leticia sampling. Each OTU with complete taxonomic description and category classification (Human Host, Parasite Protist, Parasite Nematode, Other Animal, Other Protist, Fungi, Plant & Green Algae). **Sheet 2.** Parasite nematode and parasite protist detection per sample for Leticia sampling (1 = at least one read detected; 0 = no read detected).

**Figure S1.**  Complementary Leticia gut bacteria microbiota alpha diversity analysis. **(A)** Rarefaction curve for Leticia and Km11 samples. **(B)** Number of OTUs (richness estimator) for rarefacted dataset to the lowest read count. Difference between locations evaluated using Wilcoxon rank-sum test. ns: p > 0.05, *: p <= 0.05, **: p <= 0.01, ***: p <= 0.001, ****: p <= 0.0001.

**Figure S2.** Relative bacterial family abundance for Amazonian and non-Amazonian datasets with each bar summarizing the total of samples per dataset. Families with < 1% abundance were merged into one group.

**Figure S3.**  Complementary gut bacteria microbiota alpha diversity analysis in Amazonian and non-Amazonian datasets. **(A)** Rarefaction curve for all the datasets. **(B)** Number of OTUs (richness estimator) for rarefacted datasets to the lowest read count. Letters reflect grouping and differences between datasets evaluated using Tukey HSD test.
